# Nursing students’ attitudes towards the use of digital technology in the healthcare of older adults- a cross-sectional study in Norway and Sweden

**DOI:** 10.1186/s12912-023-01600-6

**Published:** 2023-11-14

**Authors:** Ann-Chatrin Linqvist Leonardsen, Camilla Hardeland, Jenny Hallgren, Ingrid Femdal, Dip Raj Thapa, Ann Karin Helgesen, Carina Bååth, Liv Halvorsrud, Vigdis Abrahamsen Grøndahl, Catharina Gillsjö

**Affiliations:** 1 Faculty of Health, Welfare and Organization, Ostfold University College, Ostfold University College, Postal box (PB) 700, Halden, NO-1757 Norway; 2 Department of Anesthesia, Ostfold Hospital Trust, Postal box code 300, Grålum, 1714 Norway; 3 School of Health Sciences, University of Skövde, Postal box code 408, Skövde, 541 28 Sweden; 4 Faculty of Health, Science, and Technology, Department of Health Sciences, Karlstad University, Karlstad, SE-651 88 Sweden; 5 Faculty of Health Sciences, Department of Nursing and Health Promotion, Oslo Metropolitan University, Pilestredet 32, Oslo, 0166 Norway; 6 College of Nursing, University of Rhode Island, Kingston, RI USA

**Keywords:** Attitudes, Digital technology, Nursing students, Older adults, Questionnaire

## Abstract

**Background:**

Implementation of digital technology has been suggested as a potential solution to future healthcare challenges. Healthcare personnel’s attitudes are important in the acceptance and implementation of digital technologies.

**Aim:**

The aims of this study were to (1) translate and validate two different questionnaires to Norwegian and Swedish respectively, and then (2) use these to examine nursing students’ attitudes towards digital technology in healthcare, as well as their attitudes towards older adults’ abilities to use digital technology.

**Design:**

Cross-sectional.

**Methods:**

A web-based questionnaire was distributed in first year nursing students in a Norwegian and a Swedish university college, respectively. The questionnaire consisted of the short form of the ‘Information Technology Attitude Scales for Health (ITASH)’ and the ‘Attitudes Towards Older Adults Using Digital technology (ATOAUT-11)’ questionnaire. The questionnaires were translated and validated in both countries. Frequencies, Student’s t-test, and one-way ANOVA were used to analyze the data.

**Results:**

In total 236 students responded to the questionnaire in the period September 2022 to April 2023. Students mainly reported positive attitudes towards digital technology use in general. They most agreed with the items ‘Using digital technology devices makes my communication with other health professionals faster’, ‘The sort of information I can get from the digital technology devices helps me give better care to patient’, and ‘Digital technology skills are becoming more and more necessary for healthcare professionals’. However, they reported more negative attitudes towards older adults using digital technology. They most agreed with the items ‘One needs a lot of patience to explain to an older adult how to use digital technologies’, ‘It’s hard to explain to older adults how to use digital technology’, ‘Using digital technology is harder for most older adults’, and ‘Most older adults fear using digital technology because they fear of being scammed or cheated’.

**Conclusion:**

The ITASH and the ATOAUT-11 is appropriate for use in a Norwegian and Swedish setting. Even if nursing students are positive to digital technology in healthcare in general, they are sceptical to older adults using digital technology. This may impact on their attitudes to using digital technology in the healthcare of older adults. These aspects need emphasis when revising nursing education curricula focusing on developing technological competencies in nursing, and gaining knowledge regarding older adults’ use of digital technology.

**Supplementary Information:**

The online version contains supplementary material available at 10.1186/s12912-023-01600-6.

## Introduction

Internationally, registered nurses (RN) make an essential contribution to universal health coverage, emergency preparedness and response, patient safety, and the delivery of integrated and coherent person-centred care [1]. However, by the year 2030, the world faces a nursing shortage estimated to 5.7 million [2]. One of the reasons is the increasing amount of older adults, persons aged 60 years and older, with multiple health problems and complex healthcare needs, resulting in an increased demand for healthcare services [3]. The digitalization of healthcare services and the emphasize of digital courses in the education of nurses have been introduced as an initiative to address these challenges to preserve quality care [4].

Internationally, the increase of older adults and decrease in workforce is a challenge for the health and social care authorities [3, 5]. The aging process is often associated with multiple healthcare problems with extensive and complex care needs [6, 7]. The ongoing transfer of care from hospital to home is an additional challenge [8, 9]. Due to these circumstances the implementation of digital technology become increasingly important to provide a sustainable quality care [10]. In both Norway and Sweden, the use of digital solutions is rapidly increasing in the provision of healthcare in both regions and municipalities [11–13]. In addition, most patients in need of healthcare are over 65 years old. Both the Norwegian and the Swedish government have invested in safety preserving digital technology such as digital safety alarms, digital supervision, nurse call systems, and digital medicine dispensers and sensors [10, 14]. However, implementing this in health and social care for older adults may be challenging [10].

Central aspects influencing older adults’ use of digital technology are their attitudes, skills, abilities, self-efficacy, confidence, willingness to use the technology and curiosity. Other issues are design, usefulness, sense of safety and integrity. Furthermore, the information provided and available education matters [15–17]. Digital technology has been shown to empower and support social inclusion and counteract sense of loneliness [18–21]. However, studies also claim that older adults are vulnerable to loss of participation and social contacts when using digital technology in health and social care [22, 23].

The impact of digital technology is a professional issue relating to nursing care delivery, practice, education and research [4, 24]. For example, how nurses receive and review diagnostic information, make clinical decisions, communicate with patients and their relatives, and implement clinical interventions will be affected by the integration of digital technology into nursing practice [25, 26]. As such, the American Association of Colleges of Nursing underlines that core competencies for nursing education include informatics, social media, and emergent technologies and their impact on decision making and quality [27]. The significance of informatics and emergent technologies in nursing is also stated in both Norway and Sweden, being one of six core competences in the nursing curricula [28, 29]. Consequently, the digital skills that nurses need, reach beyond understanding how the digital technology work, including an ability to instruct patients in their use [30]. A digital transformation of the nursing profession may in addition lead to a need for reforming the nursing education [31]. Also, the lack of studies on technological literacy in nursing education has been highlighted [32].

The Organisation for Economic Co-operation and Development (OECD) states that digital transformation in the health sector requires adaptive change in human attitudes and skills, as well as of legal frameworks and the organization of work [33]. The literature shows that healthcare personnel’s knowledge is a crucial determinant of whether digital technology is adopted or not [34, 35]. Moreover, healthcare personnel’s attitudes are important in the acceptance and implementation of technologies [36, 37]. Some studies have shown that barriers for nurses using digital technology are lack of digital experience, confidence, competency and education [38, 39]. Warshawski et al. [40] found that nurses and nursing students had positive attitudes and felt competent toward digital technology use in clinical practice. However, students’ self-efficacy using digital technology was significantly higher than that of nurses. Also, Lee and Clarke [41] found that nursing students had positive attitudes toward the influence of digital technology on care values and the teaching of digital technology skills.

To our knowledge, no studies have examined nursing students’ attitudes to digital technology combined with their attitudes towards older adults’ use of digital technology. Through literature searches, the questionnaires ‘Information Technology Attitude Scales for Health’ (ITASH) [41] and the ‘Attitudes Towards Older Adults Using Digital technology’ (ATOAUT-11) [30] questionnaires were identified. However, these had not been translated to either Norwegian or Swedish [42].

The aims of this study were to (1) translate and validate two different questionnaires to Norwegian and Swedish respectively, and then (2) use these to examine nursing students’ attitudes towards digital technology in healthcare, as well as their attitudes towards older adults’ abilities to use digital technology.

## Methods

### Study design

The study had a cross-sectional design. The study adheres to the Strengthening the Reporting of Observational Studies in Epidemiology (STROBE) guidelines [43].

### Setting

The study was conducted in two universities, in Norway and in Sweden. The universities have 240 and 87 first semester nursing students respectively. Nettskjema.no, a survey solution developed and hosted by the University of Oslo, was used for data collection. The students were invited to complete the questionnaire by email, and in person in the classroom. The questionnaire included information about the study, assuring principles of confidentiality. Submitted questionnaires were interpreted as willing consent to participate. Recruitment and data collection were handled in the period September 2022 to April 2023.

### Participants

We used a purposive sampling strategy aiming to include a specific group of individuals, namely students. As such, all nursing students from the first semester of nursing education (*n* = 327) during the schoolyear of 2022/2023 from both universities were invited. There were no inclusion or exclusion criteria.

The students were informed out the study in the classroom and through the digital learning platform Canvas® by one of the researchers from Norway and Sweden, respectively. In Norway, the researcher had no educational responsibility for the students, but in Sweden the researcher was also a lecturer. Reminders were also given through Canvas ® and by other lecturers at two time points during class.

### Data sources

The questionnaire consisted of two validated tools:

1. The short form of the ITASH [41, 44], which consists of four scales: (1) Care Value of digital technology (four items), (2) Training of digital technology skills (six items), (3) Digital technology Confidence (six items), and (4) Workload value of digital technology (five items). The items are scored on a four-point Likert scale, where 1 = strongly disagree, and 4 = strongly agree.
2. The ATOAUT-11 questionnaire [45], which consists of 11 items relating to healthcare personnels’ attitudes toward e.g. older adults’ abilities to use digital technology, ease of use and perceived benefit, fear, anxiety, and self-efficacy. The items are scored on a six-points Likert scale where 1 = totally disagree to 6 = totally agree.

In addition, the demographic variables of students’ sex and age, and their years of experience from work in healthcare before nursing education, were collected. The questionnaire contained a total of 35 items.

### Translation process

The ITASH and the ATOAUT-11 were translated “forward and backward” in-line with recommendations from Brislin [46]. Firstly, the questionnaires were translated from English into Norwegian by two independent researchers with Norwegian as their mother-tongue, and fluent in English. The two different versions were compared and collated into one. Then, a researcher with English as her mother-tongue translated the questionnaires back to English. Finally, the research group compared and evaluated the two different English versions in relation to semantic, idiopathic and conceptual equivalence. The English version was then similarly translated into Swedish by two persons, one researcher and one administrative employee with Swedish as a mother-tongue and both speaking and writing English fluently. The two versions were compared and collated into one. The questionnaires were then translated back into English by a researcher speaking and writing English fluently.

### Validation

Lastly, the Swedish translation and Norwegian translations were assessed for face and content validity by the research group consisting of both Norwegian (*n* = 6) and Swedish (*n* = 4) researchers, registered nurses, PhD, well experienced with questionnaire validation. As such, the questionnaires were shared with colleagues in the two universities respectively, and potential inputs were encouraged. In addition, the questionnaires were discussed in mutual meetings.

### Quantitative variables

Frequencies were used to present characteristics of the study sample. Since students’ age and years of experience were not normally distributed, the results are presented as range and median (interquartile range). Responses to the questionnaires were handled as continuous variables, and not ordinal, as in previous studies using these tools [41, 44, 45, 49]. These results were summarized by their mean and standard deviation (SD), due to data being normally distributed. Analyses were done to compare results from Norway and Sweden respectively, and to assess potential associations between students’ gender, age and experience and their responses to the questionnaires.

### Statistical methods

Data were analyzed using the Statistical Package for the Social Sciences (SPSS), version 27 [47]. The internal consistency of the two questionnaires respectively, was analyzed using Cronbach’s Alpha. Groups were compared using the Students’ t-test, since data were normally distributed. Associations were explored using one-way analysis of variance (ANOVA). A significance level of *p* < .05 was assumed. There were no missing data.

## Results

### Participants

In total, 236 students (72.2%) responded to the questionnaire. This equals 68.3% of the Norwegian students, and 87.8% of the Swedish students.

### Descriptive data

Table 1 gives an oveview of the respondents' characteristics (see Table 1).

**Table 1:** Gives an overview of respondents’ characteristics

Respondents’ characteristics (*N* = 236)	
Norway, *n* (%)	164 (69.5)
Sweden, *n* (%)	72 (30.5)
Female, *n* (%)	212 (89.1)
Age	
Range, years	18–54
Median (IQR)*	22 (19–20)
Previous experience from healthcare, *n* (%)	169 (71)
Years of experience, range	0–17
Median (IQR)	4 (1–6)

*IQR = interquartile range

Regarding previous experience from healthcare, students mainly had worked as assistants in assisted living facilities for older adults, or in home care services.

### Main results

Students most agreed with the items ‘Using digital technology devices makes my communication with other health professionals faster’ (mean score 3.6), ‘The sort of information I can get from the digital technology devices helps me give better care to patients’ (mean score 3.4), and ‘Digital technology skills are becoming more and more necessary for healthcare professionals’ (mean score 3.4). They least agreed on the items ‘Using digital technology devices is more trouble than it’s worth’ (mean score 1.8), ‘I sometimes feel very intimidated by the thought of using digital technology devices’ (mean score 2), and ‘Where I work, digital technology devices make staff less productive’ (mean score 2.1). There were eight significant differences between Norwegian and Swedish students respectively (see Table 2).

**Table 2:** Responses to the Information Technology Attitude Scales for Health (*N* = 236)

	Norway (*n* = 164) Mean (SD)	Sweden (*n* = 72) Mean (SD)	Total (*n* = 236) Mean (SD)	*p* value
Using digital technology devices is helping to improve patient/client care	3.1 (0.6)	3.3 (0.5)	3.2 (0.6)	0.80
The sort of information I can get from the digital technology devices helps me give better care to patients	3.4 (0.6)	3.3 (0.5)	3.4 (0.6)	0.004**
Using digital technology devices makes my communication with other health professionals faster	3.7 (0.5)	3.3 (0.7)	3.6 (0.6)	0.001**
I believe digital technology devices can help us deliver individualized care	3.2 (0.7)	3.2 (0.6)	3.2 (0.7)	0.86
I feel I need more training to use the digital technology devices properly	2.9 (0.8)	3.2 (0.7)	3 (0.8)	0.81
I would like to have ongoing training to help me improve my digital technology skills	2.9 (0.7)	3.3 (0.6)	3 (0.7)	0.98
Digital technology skills are becoming more and more necessary for healthcare professionals	3.4 (0.6)	3.5 (0.6)	3.4 (0.6)	0.39
In order to be successful in my career I need to be able to work with digital technology devices	3.3 (0.7)	3.2 (0.7)	3.2 (0.7)	0.62
Using digital technology devices helps to increase professionals’ knowledge base	3.2 (0.6)	3.2 (0.7)	3.2 (0.6)	0.10
I would like to know more about digital technology devices generally	3.1 (0.7)	3.3 (0.6)	3.1 (0.7)	0.83
I lack confidence in my general digital technology skills	2 (0.7)	2.5 (0.9)	2.2 (0.8)	< 0.001**
I generally feel confident working with digital technology devices	3 (0.9)	2.8 (0.8)	2.9 (0.7)	0.004**
I have all the general digital technology skills I need for my job	3 (0.6)	2.4 (0.9)	2.8 (0.8)	0.001**
I am easily able to learn new digital technology skills	3.3 (0.6)	3.1 (0.8)	3.2 (0.7)	0.35
I am often unsure what to do when using digital technology devices	2.1 (0.7)	2.4 (0.8)	2.2 (0.7)	0.008**
I sometimes feel very intimidated by the thought of using digital technology devices	2 (0.8)	2.3 (0.9)	2 (0.8)	0.03*
Using digital technology devices is more trouble than it’s worth	1.8 (0.6)	2.1 (0.9)	1.8 (0.7)	0.001**
Where I work, digital technology devices make staff less productive	2.1 (0.7)	2.3 (0.8)	2.1 (0.7)	0.05
I feel there are too many digital technology devices around now	2.3 (0.8)	2.3 (0.9)	2.3 (0.8)	0.72
I think we are in danger of letting digital technology devices take over	2.4 (0.9)	2.5 (0.9)	2.4 (0.9)	0.70
Time spent on digital technology devices is out of proportion to its benefits	2.2 (0.7)	2.4 (0.8)	2.3 (0.7)	0.3

Information Technology Attitude Scales for Health, where 1 = strongly disagree, 2 = disagree, 3 = agree, and 4 = strongly agree. Student’s t-test. *p* < .05 = statistically significant. *= significant at a 0.05 level. **=significant at a 0.01 level

Students most agreed with the items ‘One needs a lot of patience to explain to an older adult how to use digital technologies’ (mean score 4.8), ‘It’s hard to explain to older adults how to use digital technology’ (mean score 4.6), ‘Using digital technology is harder for most older adults’ (mean score 4.6), and ‘Most older adults fear using digital technology because they fear of being scammed or cheated’ (mean score 4.6). Students least agreed on the items ‘Most older adults can use digital technology just as well as younger adults’ (mean score 2.8). No significant differences between countries were found (see Table 3).

**Table 3:** Responses to the Attitudes Towards Older Adults Using Technology (ATOAUT-11) (*N* = 236)

	Norway (*n* = 164) Mean (SD)	Sweden (*n* = 72) Mean (SD)	Total (*n* = 236) Mean (SD)	*p*-value
It’s hard to explain to older adults how to use digital technology	4.5 (1)	4.6 (1.1)	4.6 (1)	0.38
Most older adults can use digital technology just as well as younger adults	2.7 (1.3)	3 (1.4)	2.8 (1.3)	0.71
Most older adults have less access to digital technology	4 (1)	4.6 (1.1)	4.2 (1.1)	0.32
Most older adults do not see the benefits of using digital technology	4.1 (1.1)	4.3 (1.2)	4.2 (1.1)	0.06
Using digital technology is harder for most older adults	4.6 (1)	4.7 (0.9)	4.6 (1)	0.44
Most older adults can give useful feedback about new digital technologies	3.7 (1.1)	3.8 (1.3)	3.7 (1.2)	0.75
Online services can be used by adults of any age (for example online banking or government services)	4.1 (1.4)	4.1 (1.3)	4.1 (1.3)	0.07
Most older adults fear using digital technology because they believe they will break or ruin something	4.3 (1.2)	4.6 (1.1)	4.4 (1.2)	0.30
Most older adults are not interested in learning about using new digital technologies	4.1 (1.1)	4 (1.2)	4 (1.2)	0.72
One needs a lot of patience to explain to an older adult how to use digital technologies	4.9 (1)	4.7 (1)	4.8 (1)	0.93
Most older people fear using digital technology because they fear of being scammed or cheated	4.5 (1.2)	4.8 (1.1)	4.6 (1.2)	0.23

Attitudes Towards Older Adults Using Digital technology (ATOAUT-11) where 1 = totally disagree, and 6 = totally agree. Student’s t-test. *p* < .05 = statistically significant

### Other analyses

A one-way analysis of variance (ANOVA) between respondents’ age and years of experience from working in health and social care services, and their responses to the ITASH and the ATAUT-11 respectively, showed three positive significant associations, meaning that the values of one variable tend to increase as the values of the other variable increase. Associations were identified between age and ‘I have all the general digital technology skills I need for my job’ (*F* = + 1.7, *p* = .02), and ‘Using digital technology is harder for most older adults’ (*F* = + 1.6, *p* = .03), between ‘Previous experience from healthcare services’ and ‘Using digital technology devices is helping to improve patient/client care’ (*F* = + 1.9, *p* = .004) and ‘I would like to have ongoing training to help me improve my digital technology skills’ (*F* = + 1.6, *p* = .04).

The Cronbach’s Alpha of the ITASH was 0.67, and for the ATOAUT-11 it was 0.69.

## Discussion

### Key results

To our knowledge, this is the first study examining nursing students’ attitudes towards both digital technology in general and towards older adults using digital technology combined. The results indicate that nursing students both in Norway and Sweden are positive to digital technology use in general, but more critical to older adults using digital technology, irrespective of age or previous working experience within healthcare. This could imply that they themselves are critical to using digital technology in the healthcare to older adults. Keeping the future healthcare challenges and national priorities in mind, this will be a potential barrier for implementing digital technology in health and social care.

### Limitations

A strength of this study is the use of validated questionnaires, which were rigorously translated [46]. The final versions of the questionnaires were assessed by experienced clinicians and researchers, hereby supporting the face and content validity.

The internal consistency of the questionnaires, as measured by Cronbach’s Alpha, of the ITASH was 0.67, and of the ATOAUT-11 was 0.69, and barely acceptable [48]. This is also lower than in previous studies [45, 49]. One reason may be that the respondents were too homogeneous [48]. We could also have done sub-scale analyses of the questionnaires [41, 44]. Due to that the responses to the questionnaires provided information to reach the study aims, we chose not to do this.

We did not conduct any sample size calculations, still the number of participants coheres with sample size suggestions for cross-sectional studies [42].

Students were invited/reminded about the study by their lecturer. This may have represented a selection bias. However, it was stated that participation was voluntary, and that non-participation would not have any negative consequences for the student. The recruiters did not have access to the data, including the students’ background information.

Whether the results are generalizable to other settings may be questioned. The sample consisted of few male respondents, which is transferable to the nursing student population in total. However, including nursing students with a range of age and years of previous experience from working in health and social care before entering the nursing education, and from two university colleges and countries, support the transferability of our findings.

### Interpretation

Nursing students in the current study agreed most with the items ‘Using digital technology devices makes my communication with other health professionals faster’, and ‘The sort of information I can get from the digital technology devices helps me give better care to patients’, from the ‘care value’ scale, and ‘Digital technology skills are becoming more and more necessary for healthcare professionals’ from the ‘Training of digital technology skills’ scale of the ‘Information Technology Attitude Scales for Health’ (ITASH) questionnaire [44]. These findings are in-line with findings in the study of Lee and Clarke [41], and indicate that nursing students acknowledge the digital technology care value and the importance of digital technology training in nursing.

Additionally, nursing students in our study agreed least on the item ‘I sometimes feel very intimidated by the thought of using digital technology devices’ from the ‘Digital technology confidence’ scale, as well as the items ‘Using digital technology devices is more trouble than it’s worth’, and ‘Where I work, digital technology devices make staff less productive’ from the ‘Digital technology workload value’ scale of the ‘Information Technology Attitude Scales for Health’ (ITASH) questionnaire [41, 45]. Hence, they reported positive attitudes to digital technology use in their work as a nurse. Other studies have also found that nursing students have positive attitudes towards technology use in general [49, 50]. However, studies have also shown that nursing students prefer traditional nursing activities rather than technology-based healthcare [51, 52]. Both nationally and internationally, digital technology is emphasized as a core competency for nurses [27–29]. It is unlikely that nurses in the future are allowed to not use digital technology. Hence, digital technology needs emphasis in the nursing curricula.

Nursing students in the current study agreed that it requires patience and is hard to explain to an older adult how to use digital technologies. Moreover, they assumed that it will be harder for most older adults to learn using digital technology, and also that they fear to use it. There is a lack of studies exploring nursing students’ attitudes towards digital technology use in older adults, however similar findings have been reported in studies of health and social care personnel and nurses [45, 53, 54]. These studies indicated an association between negative attitudes towards older adults using and adopting digital technology and ageism, in means of stereotypes, prejudice, and discrimination towards a person because of their age [55]. A 2023 scoping review identified negative attitudes to older adults in nursing students [56]. Deficiency in education and experience regarding gerontology and geriatrics have been found to be associated with ageism [54–56]. In the current study we did not explore nursing students’ attitudes towards older adults, and we cannot claim such associations. Also, these findings are in contrast to studies finding that older adults frequently use digital technology [57], and they feel positively about digital technology [58–60]. However, it needs to be considered that for some older adults there are barriers such as experience of anxiety, fear and hesitancy related to the use of technology [61]. Consequently, these are important aspects to keep in mind to incorporate in the education when revising nursing curricula for the future.

In the current study, positive associations were seen between increasing age or previous work experience and nursing students’ attitudes towards digital technology and digital technology use in older adults. This means the students’ tended to be more positive both towards using digital technology and towards older adults using digital technology as they gained older and more experienced themselves. This is in contrast to findings of Ward et al. [44], who found no significant associations between age and attitudes towards digital technology, or Lee and Clarke [41] who found that nurses being older and more experienced had more negative attitudes towards digital technology than nursing students. One assumption in the current study is that students may have experienced older adults using digital technology, and that this was feasible. Also, it may be difficult to imagine older adults’ use of digital technology when lacking healthcare experience at all. However, Mannheim et al. [45] found that stereotype activation accounted more for health care professionals’ attitudes to use of digital technology in older adults, than did the experience of working with older patients or the professionals’ age. Some studies have found significant differences between females and males, indicating a more positive attitude towards digital technology among male university students [62, 63]. Due to the low proportion of male students in this study, we were not able to explore any differences between male and female students.

Eight significant differences between Norwegian and Swedish nursing students regarding attitudes towards digital technology were identified. The results indicated that Norwegian nursing students felt more confident in digital technology use than Swedish nursing students. We have not identified other studies assessing cross-national differences. Both Norwegian and Swedish national guidelines and reports focus on digital technology competence in nursing [5, 6, 9, 10]. Hence, we cannot find any reasonable explanation for these differences between countries. Since the nursing students responded to the questionnaire in the beginning of the first semester, the difference might be related to differences in high school in the two countries. A second data collection is planned at the end of the education in both countries as well. The students’ responses here will be a potential indicator for differences between the nursing curricula related to digital competence.

The adoption of digital technology is necessary to contribute to the new digital-based models of care entering the healthcare sector globally [64, 65]. Consequently, digital competence is stated as an overall important part in health care professionals’ clinical competence, especially in times with continuously changes in healthcare practice [66]. Nevertheless, a 2019 systematic review concluded that despite increasing concern on the use of digital technology, its integration in nursing education has not been given significant attention [67].

## Conclusions

The results in this study help to fill the noted gap in research regarding nursing students and nursing education, and the digital transformation. The results indicate that even if nursing students are positive to digital technology in healthcare in general, they are more sceptical to older adults’ using digital technology. To increase nursing student readiness in the digital transformation, the nursing education curricula should include education regarding use of digital technology in provision of health care to older adults specifically. Ageism also needs to be addressed in relation to older adults and digital technology.

Future studies should include information about nursing students’ attitudes to older adults, to detect and hopefully prevent potential ageism. Future studies should also focus on potential explanations to the cross-country differences in attitudes.

### Electronic supplementary material

Below is the link to the electronic supplementary material.

Supplementary Material 1: STROBE Statement?checklist of items that should be included in reports of observational studies

## Data Availability

Materials are available from the corresponding author upon request.
